# Supplementary material for: RGT: a toolbox for the integrative analysis of high throughput regulatory genomics data
Source: BMC Bioinformatics. 2023 Mar 6;24:79. doi: 10.1186/s12859-023-05184-5 (PMC9990262; doi:10.1186/s12859-023-05184-5)
Supplement: Supplementary file 1 — Additional file 1. Fig. S1. An example pipeline for the analysis of ATAC-seq data; Fig. S2. Schematics of regions versus regions tests in RGT-viz; Fig. S3. Schematics of plotting tools in RGT-viz; Fig. S4. An screenshot showing the results of motif enrichment analysis for TF PU.1 (also known as Spi1); Table S1. ChIP-seq data used in the dendritic cell development case study of RGT-viz and Table S2. Statistical results of intersection test between PU.1 and IRF8 ChIP-seq peaks across different cell types. [file 12859_2023_5184_MOESM1_ESM.pdf]

RGT: a toolbox for the integrative analysis of high throughput regulatory genomics data

Zhijian Li[1,2,*+], Chao-Chung Guo[1,2,+], Fabio Ticconi[1,2], Mina Shaigan[1,2], Julia Gehrmann[1,2], Eduardo Gadde Gusmao[1,2], Manuel Allhoff[1,2], Martin Manolov[1,2], Martin Zenke[3,4,5], and Ivan G. Costa[1,2,*]

[1]Institute for Computational Genomics, RWTH Aachen University, Medical Faculty, 52074 Aachen, Germany
[2]Joint Research Center for Computational Biomedicine, RWTH Aachen University Hospital, 52062 Aachen, Germany
[3]Department of Cell Biology, Institute of Biomedical Engineering, RWTH Aachen University Medical School, 52062 Aachen, Germany
[4]Helmholtz Institute for Biomedical Engineering, RWTH Aachen University, 52062 Aachen, Germany
[5]Department of Hematology, Oncology, Hemostaseology, and Stem Cell Transplantation, Faculty of Medicine, RWTH Aachen University, 52062 Aachen, Germany
[+]Contributed equally to this work
[*]Corresponding authors: zhijian.li@rwth-aachen.de; ivan.costa@rwth-aachen.de

March 1, 2023

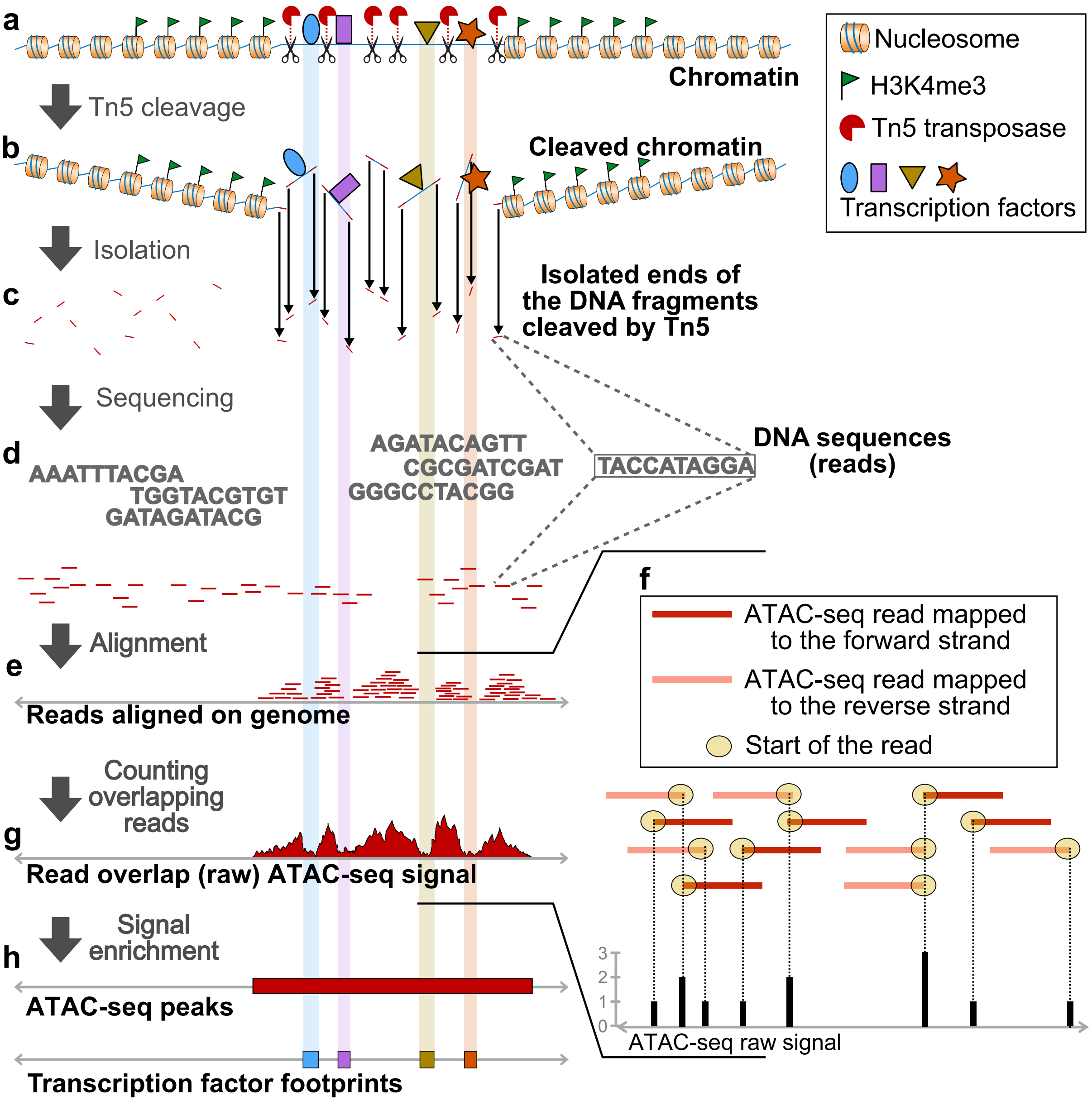

Supplementary Fig. 1. An example pipeline for the analysis of ATAC-seq data. Similar to ChIP-seq data, the first step after obtaining the sequencing data is to perform the alignment. Based on the aligned reads, a genome-wide signal is created by counting overlapping reads on the start site. Next, chromatin-accessible regions are detected by peak calling. Finally, transcription factor footprints are identified by searching for footprint-like Tn5 cleavage patterns.

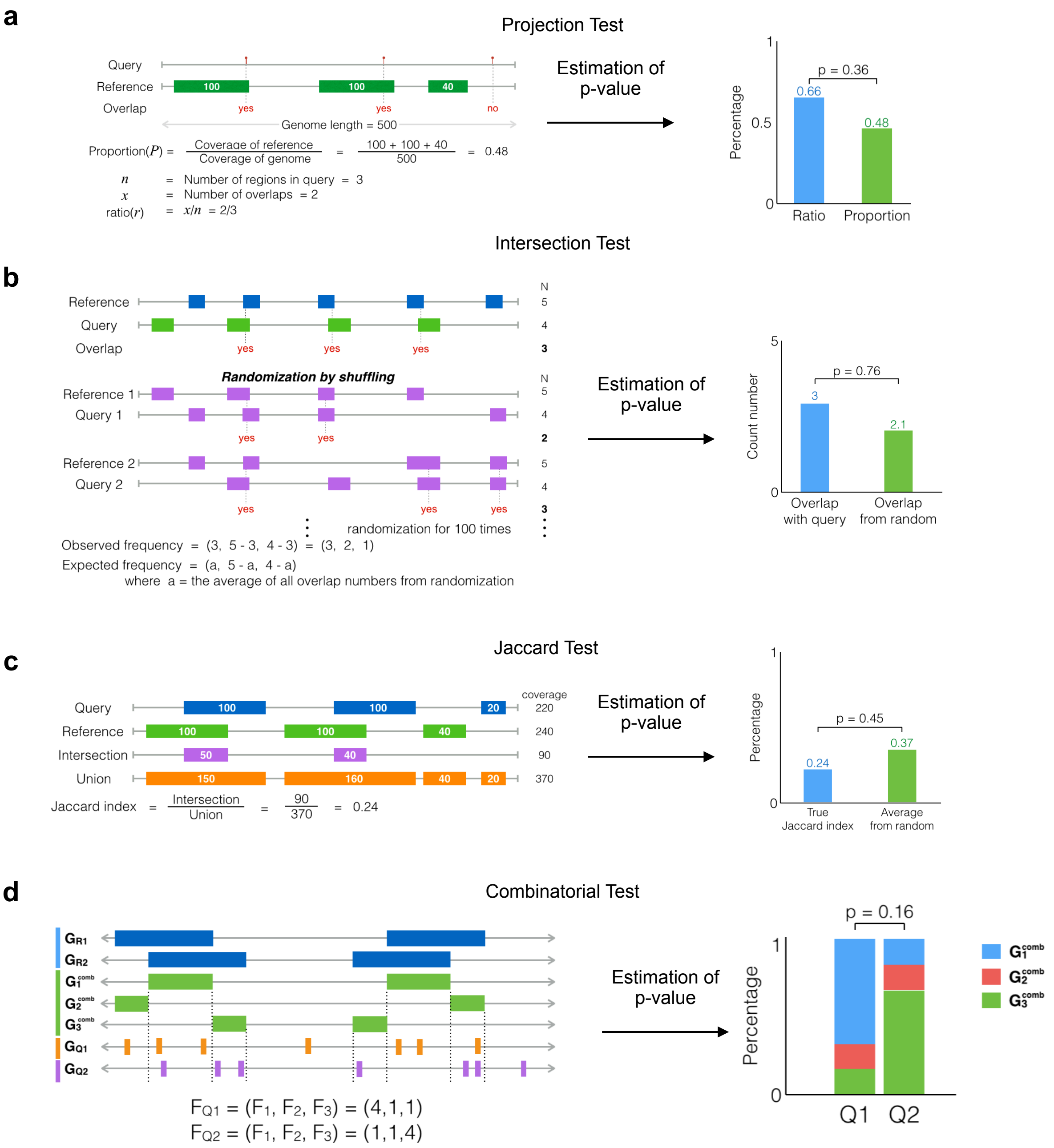

Supplementary Fig. 2. Schematics of regions versus regions tests in RGT-viz. a, In the projection test, the query regions are converted into points by their mid-points, then the number of overlaps between reference and point-wise queries are counted. All relevant statistics are calculated such as background proportion (P), which considers the overlap of the reference regions to the original genome, and ratio (r), which considers the overlap between the query and reference region sets. **b,** The intersection test counts the overlaps between query and reference as observed frequency. Randomization is performed by re-sampling region sets from the intersection of both query and reference genomic region sets. This distribution is used to estimate an empirical p-value. **c,** Jaccard test calculates the amount of the overlap, which is the ratio of intersection to the union. Randomization is also performed at least 1000 times. The true Jaccard index is compared with the average of random Jaccard indexes graphically and used to estimate empirical p-values. **d,** The combinatorial test counts the overlaps between each query and each combination of references. These numbers form a profile for each query and can also be compared with one another graphically and using a chi-square test.

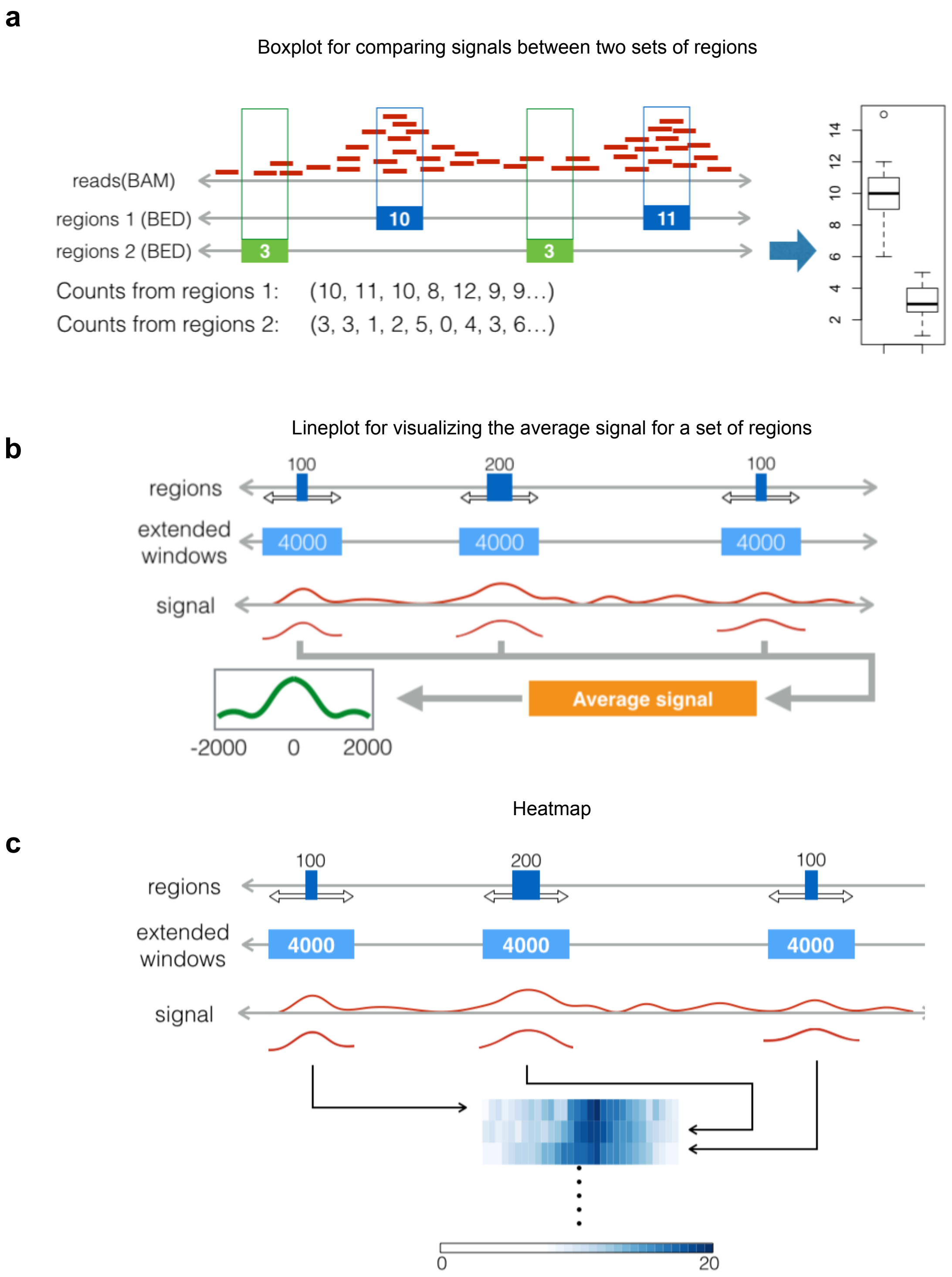

Supplementary Fig. 3. Schematics of plotting tools in RGT-viz. **a,** The boxplot compares the reads from one BAM file between two sets of regions represented as BED files. The numbers of reads which fall into the regions of interest are counted, normalized, and compared. **b,** The line plot visualizes the signal of a set of regions. The regions are extended from their midpoint to a window of the same size. According to these windows, the signals on the genome are averaged to form the line plot. **c,** In contrast to the line plot, the heatmap displays all the read counts over the region (x-axis) over distinct regions (y-axis) in a two-dimensional matrix. Colors correspond to the number of reads.

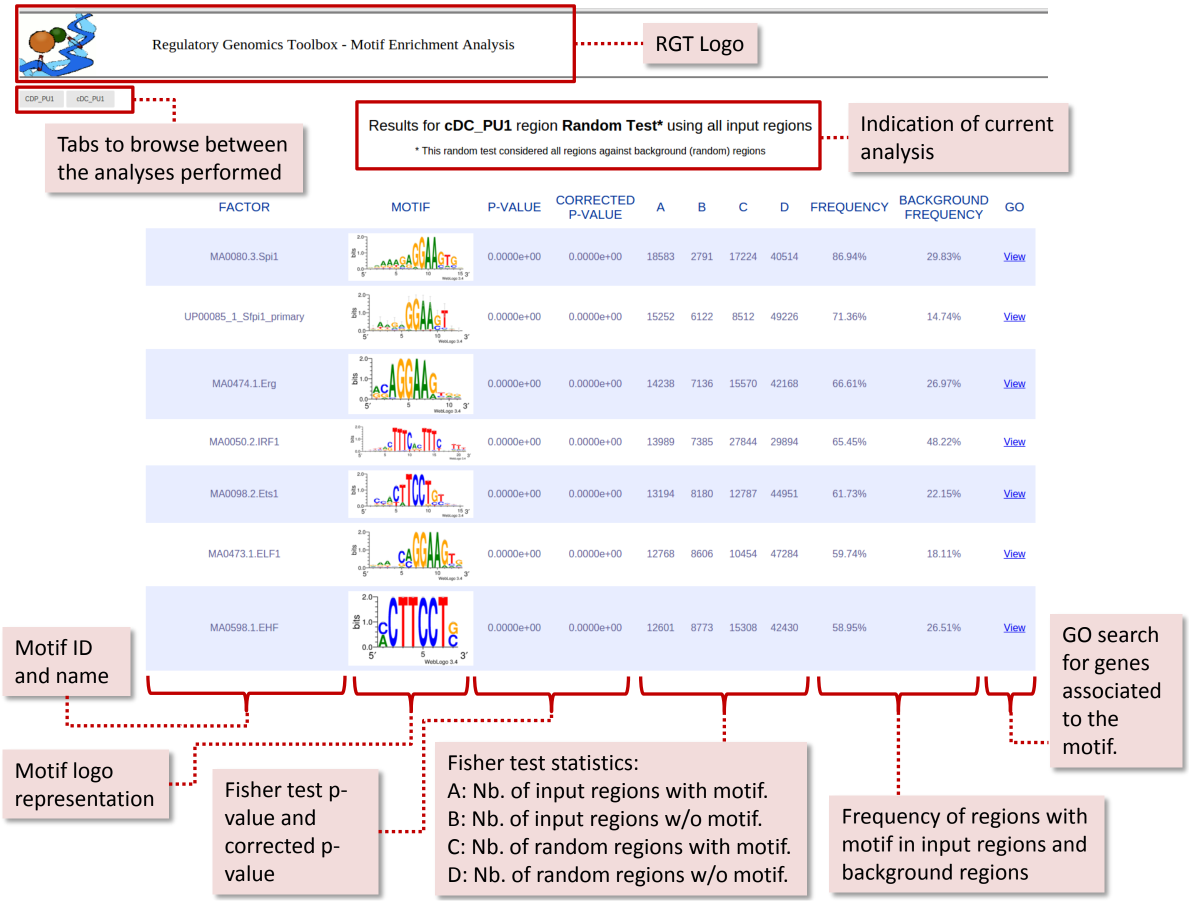

Supplementary Fig. 4. An screenshot showing the results of motif enrichment analysis for TF PU.1 (also known as Spi1). Here, we performed motif matching and enrichment analysis on the PU.1 peaks of the dendritic cell types CDP and cDC. The first two columns showed the motif name and logo found in the repositories. Next, the raw and corrected p-values are shown. The A and B columns represent the number of input regions with at least 1 occurrence of and with no occurrence of that transcription factor. The C and D columns represent the number of background regions with at least 1 occurrence of and with no occurrence of that transcription factor. As we can observe, as expected, PU.1 motifs were ranked in the top (UP00085_1_Sfpi1_primary and MA0080.3.Spi1). Furthermore, we observed that other transcription factors were also enriched in these regions, such as Erg, IRF1, Ets1, ETF1, EHF, and many others. These transcription factors are putative co-binding or regulatory partners of PU.1 and are connected with PU.1 in its regulatory network within the cDC cell type.

Table 1: ChIP-seq data used in the dendritic cell development case study of RGT-viz

Transcription factor or histone modification	cell type	GEO accession number
IRF8	cDC	GSE198651
IRF8	pDC	GSE198651
PU.1	MPP	GSE57563
PU.1	CDP	GSE57563
PU.1	cDC	GSE57563
PU.1	pDC	GSE57563
H3K4me1	MPP	GSE57563
H3K4me1	CDP	GSE57563
H3K4me1	cDC	GSE57563
H3K4me1	pDC	GSE57563
H3K4me3	MPP	GSE64767
H3K4me3	CDP	GSE64767
H3K4me3	cDC	GSE64767
H3K4me3	pDC	GSE64767
H3K9me3	MPP	GSE64767
H3K9me3	CDP	GSE64767
H3K9me3	cDC	GSE64767
H3K9me3	pDC	GSE64767
H3K27me3	MPP	GSE64767
H3K27me3	CDP	GSE64767
H3K27me3	cDC	GSE64767
H3K27me3	pDC	GSE64767
H3K27ac	MPP	GSE73143
H3K27ac	CDP	GSE73143
H3K27ac	cDC	GSE73143
H3K27ac	pDC	GSE73143

Table 2: Statistical results of intersection test between PU.1 and IRF8 ChIP-seq peaks across different cell types. Rows are sorted based on χ^2 statistics.

Ref. name	Query name	Ref. number	Query number	Intersect.	Average intersect.	χ^2-stat.	Positive association p-value	Negative association p-value
cDC_PU1	cDC_IRF8	20054	34003	13973	6538	4489	0	1
CDP_PU1	cDC_IRF8	20237	34003	14003	6574	4438	0	1
pDC_PU1	cDC_IRF8	21050	34003	14307	6757	4367	0	1
MPP_PU1	cDC_IRF8	6212	34003	4412	1163	3359	0	1
pDC_PU1	pDC_IRF8	21050	6467	4137	1478	2100	0	1
CDP_PU1	pDC_IRF8	20237	6467	4078	1494	1979	0	1
cDC_PU1	pDC_IRF8	20054	6467	4066	1497	1955	0	1
MPP_PU1	pDC_IRF8	6212	6467	1745	848.5	351.3	5.1e-77	1

Table 3: Comparison of comprehensiveness between RGT and other tools that provide similar functionalities.

	RGT	bedtools	Deeptools	pyDNase	GenomicRegions
Program language	Python	C	Python	Python	R
Command-line interface	✓	✓	✓	✓	✗
Programming interface	✓	✗	✗	✗	✓
Interval algebra	✓	✓	✗	✗	✗
Statistical tests	✓	✗	✗	✗	✗
Visualization	✓	✗	✓	✓	✗
Motif analysis	✓	✗	✗	✗	✗
ATAC/DNase-seq footprinting	✓	✗	✗	✓	✗
RNA-DNA binding prediction	✓	✗	✗	✗	✗
Differential peak calling	✓	✗	✗	✗	✗
